# The role of dendritic inhibition in shaping the plasticity of excitatory synapses

**DOI:** 10.3389/fncir.2012.00118

**Published:** 2013-04-03

**Authors:** Lital Bar-Ilan, Albert Gidon, Idan Segev

**Affiliations:** ^1^Department of Neurobiology, The Hebrew University of JerusalemIsrael; ^2^Interdisciplinary Center for Neural Computation, The Hebrew University of JerusalemIsrael; ^3^Edmond and Lily Safra Center for Brain Sciences, The Hebrew University of JerusalemIsrael

**Keywords:** synaptic plasticity, dendritic inhibition, compartmental model, dendritic cable, dendritic calcium

## Abstract

Using computational tools we explored the impact of local synaptic inhibition on the plasticity of excitatory synapses in dendrites. The latter critically depends on the intracellular concentration of calcium, which in turn, depends on membrane potential and thus on inhibitory activity in particular dendritic compartments. We systematically characterized the dependence of excitatory synaptic plasticity on dendritic morphology, loci and strength, as well as on the spatial distribution of inhibitory synapses and on the level of excitatory activity. Plasticity of excitatory synapses may attain three states: “protected” (unchanged), potentiated (long-term potentiation; LTP), or depressed (long-term depression; LTD). The transition between these three plasticity states could be finely tuned by synaptic inhibition with high spatial resolution. Strategic placement of inhibition could give rise to the co-existence of all three states over short dendritic branches. We compared the plasticity effect of the innervation patterns typical of different inhibitory subclasses—Chandelier, Basket, Martinotti, and Double Bouquet—in a detailed model of a layer 5 pyramidal cell. Our study suggests that dendritic inhibition plays a key role in shaping and fine-tuning excitatory synaptic plasticity in dendrites.

## Introduction

Excitatory synapses in hippocampal and cortical pyramidal neurons undergo long-term potentiation (LTP) when pre-before-post spiking occurs within a 10–20 ms time window, whereas the reverse temporal order induces long-term depression (LTD) (Markram et al., 1997; Bi and Poo, 1998; Debanne et al., 1998; Feldman, 2000; Sjostrom et al., 2001). The induction of LTP is thought to depend on the supra-linear summation of the local dendritic Ca^2+^ transients induced by the co-activation of the excitatory post synaptic potential (EPSP) and the back-propagating action potential, AP (Yuste and Denk, 1995; Magee and Johnston, 1997; Koester and Sakmann, 1998; Schiller et al., 1998). In contrast, when the EPSP follows the AP, a sub-linear summation of Ca^2+^ transients has been observed, leading to LTD (Koester and Sakmann, 1998). Indeed, Ca^2+^ transients have been found to be essential for the induction of LTP and LTD (Lynch et al., 1983; Malenka et al., 1992), whereas the sign of synaptic plasticity is widely believed to be determined by the amplitude and perhaps time course of the change in intracellular Ca^2+^ concentration ([Ca^2+^]_i_). A larger and more rapid increase in [Ca^2+^]_i_ gives rise to LTP, whereas smaller and slower increases in [Ca^2+^]_i_ cause LTD (Bear et al., 1987; Lisman, 1989; Artola and Singer, 1993; Neveu and Zucker, 1996; Hansel et al., 1997; Yang et al., 1999; Cho et al., 2001; Cormier et al., 2001).

The intracellular concentration of Ca^2+^ closely follows the local activity-dependent membrane potential in the dendrite (Tsien and Tsien, 1990; Berridge et al., 2000). Synaptic inhibition plays a key role in modulating the dendritic membrane potentials, thereby modulating local [Ca^2+^]_i_ (Buzsaki et al., 1996; Tsubokawa and Ross, 1996; Kanemoto et al., 2011). Therefore, inhibition is expected to strongly impact long-term synaptic plasticity. Indeed, the GABAergic agonist diazepam has been shown to block the induction of LTP (Trepel and Racine, 2000; Hu et al., 2006) and the GABAergic antagonists picrotoxin or bicuculine have been shown to facilitate the induction of LTP in both hippocampus (Wigstrom and Gustafsson, 1985; Abraham et al., 1987; Hanse and Gustafsson, 1992; Chapman et al., 1998; Huemmeke et al., 2002; Meredith et al., 2003) and neocortex (Artola et al., 1990; Castellano et al., 1993; Tropea et al., 1999; Hess, 2004; Komaki et al., 2007). In hippocampal slices, the activation of GABAergic synapses induced LTD in excitatory synapses for a pre-before-post timing protocol that would have otherwise undergone LTP (Tsukada et al., 2005). Moreover, enhanced GABAergic transmission in layer 5 pyramidal neurons in mouse prefrontal cortex reduced dendritic calcium signals associated with action potential propagation, thus increasing the threshold for synaptic spike-timing-dependent potentiation (Couey et al., 2007).

In both the hippocampus (Klausberger and Somogyi, 2008) and the neocortex (Douglas and Martin, 2009; Helmstaedter et al., 2009), individual inhibitory axons from distinct input sources target-specific dendritic subdomains, where they form multiple synaptic contacts (Markram et al., 2004). This domain-specific inhibition is expected to play a central role in controlling local non-linear dendritic processes (Miles et al., 1996; Larkum et al., 1999), as well as local synaptic plasticity. The interplay between dendritic inhibition and the plasticity of excitatory synapses is not well understood. To explore this interaction, we employed the bi-directional [Ca^2+^]_i_-dependent plasticity rule suggested by Shouval et al. (2002) (see “Materials and Methods”). By computing [Ca^2+^]_i_ in dendrites receiving excitatory and inhibitory input, we show how this local plasticity rule gives rise to a spatially non-uniform distribution of synaptic weights over the dendritic tree; the profile of this weight distribution and its sign (LTP or LTD) depend on the strength and dendritic location of the inhibitory synapses, and on the intensity and spatial spread of the excitatory input. Typically, the weight of excitatory synapses that would have undergone LTP in the absence of inhibition remained unchanged (“protected”) when these synapses were adjacent to active inhibitory synapses. At an intermediate distance from inhibition, excitatory synapses were depressed (LTD), whereas they were potentiated (LTP) when located sufficiently far from inhibition. We show that even in electrically short dendritic branches, a strategically placed inhibitory synapse may expose these three possible plasticity states of the excitatory synapses.

We next examined how synaptic plasticity is affected by different characteristic patterns of inhibitory innervations. We suggest that the activation of inhibitory connections from multiple input sources enables the fine tuning the plasticity level of excitatory synapses up to the level of controlling the weight of individual excitatory synapses. In addition, we show that the overall activity level in the neuron plays a key role in modulating the effect of inhibition on the plasticity of excitatory synapses.

## Materials and methods

### Compartmental modeling

All simulations were designed and run using NEURON 5.8 (Hines and Carnevale, 1997). Four neuron models, listed below, were used in this work. Each model contained calcium dynamics, inhibitory GABAergic synapses and excitatory AMPA/NMDA synapses undergoing [Ca^2+^]_i_-dependent plasticity as in Shouval et al. (2002). A typical simulation time of 60 s was sufficient so that no further plasticity changes were observed. The integration time step in all simulations was Δ*t* = 0.025 ms.

#### Ball-and-stick model of a dendritic branch

**Figure 1:**
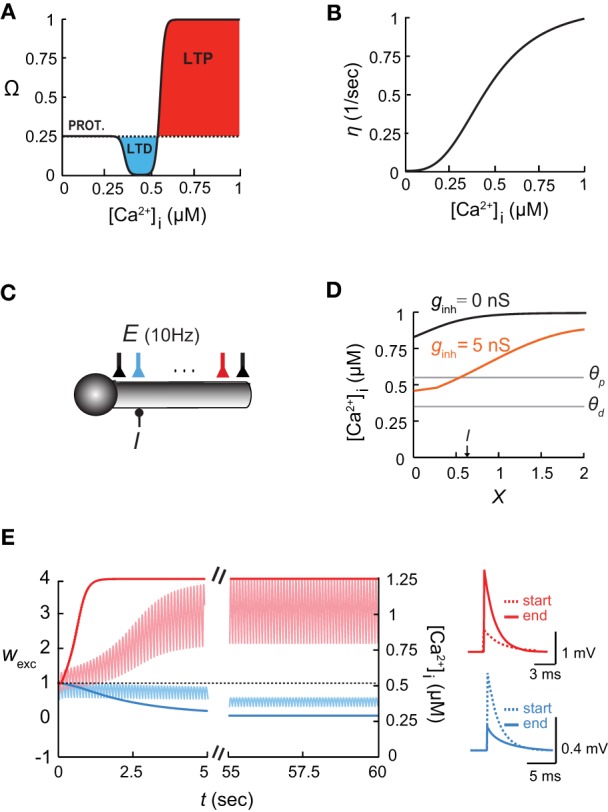
**The [Ca^2+^]_**i**_-based learning rule and the shaping of the [Ca^2+^]_i_ spatial profile in dendrites by inhibition. (A)** The plasticity function, Ω, depends on intracellular calcium concentration, [Ca^2+^]_i_. Below *θ*_d_, the synaptic weights stay at a basal level (PROT. = protected); for *θ*_d_ < [Ca^2+^]_i_ < *θ*_p_, the synapse undergoes long term depression, LTD; above *θ*_p_, it undergoes long term potentiation, LTP. **(B)** The learning rate *η* as a function of intracellular calcium (adapted from Shouval et al., 2002). **(C)** Model of a somatic compartment coupled to a 2λ long cylindrical cable with inhibitory input (*I*) at *X* = 0.6 and 21 excitatory (AMPA/NMDA-receptor-mediated) synapses activated synchronously at 10 Hz each **(E)**. Cyan and red excitatory synapses located at *X* = 0.6 and *X* = 1.6, respectively. **(D)** Intracellular calcium concentration along the dendritic cable in the absence of inhibition (black) and with a steady 5 nS inhibition (orange). The thresholds for potentiation (*θ*_p_) and depression (*θ*_d_) are marked by horizontal lines. **(E)** Left: Time course of changes in synaptic weights (*w*_exc_) and [Ca^2+^]_i_ for distal and proximal excitatory synapses [cyan and red, respectively; colors correspond to the synapses in **(C)**] throughout 60 s of simulation. Right: EPSPs of the two synapses at the beginning (dashed line) and the end (filled line) of the simulations.

**Figure 2:**
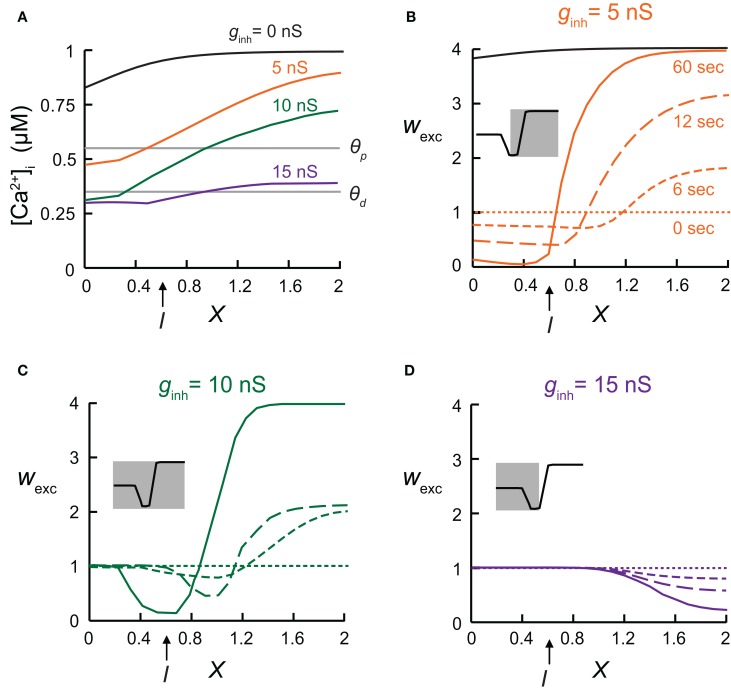
**The local synaptic plasticity rule is spatially mapped onto the dendrites. (A)** Calcium concentration, [Ca^2+^]_i_, for the model shown in Figure 1C, at various electrical distances from the soma with inhibitory conductance, *g*_GABA_, of 0 nS (black), 5 nS (orange), 10 nS (green), and 15 nS (purple). **(B–D)** Synaptic weights following 0, 6, 12, and 60 s of simulation, with the [Ca^2+^]_i_-dependent plasticity rule depicted in Figure 1. Inhibition was activated at *X* = 0.6. Insets demonstrate which of the three states comprising the plasticity rule is mapped onto the dendritic cable. **(B)** Small GABAergic conductance (*g*_GABA_ = 5 nS) switched synaptic plasticity in the vicinity of inhibition from LTP to LTD. Black—synaptic weights in the absence of inhibition after 60 s of simulation. **(C)** Intermediate inhibitory conductance (*g*_GABA_ = 10 nS) caused a strong local decrease in [Ca^2+^]_i_, leading to the absence of synaptic plasticity (*w*_exc_ = 1) in the dendritic region near the soma (between *X* = 0 and *X* = 0.3). To the right of the protected region, synapses were depressed due to lowered [Ca^2+^]_i_, and further away to the right, synapses remain potentiated. **(D)** Strong inhibition (*g*_GABA_ = 15 nS) resulted in a large protected zone whereas further away to the right excitatory synapse were depressed.

**Figure 3:**
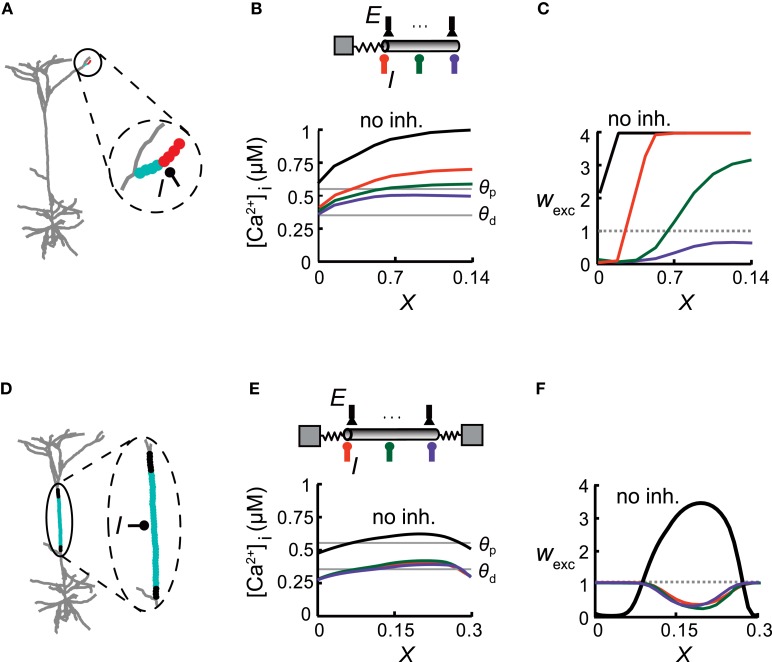
**Inhibitory impact on excitatory plasticity depends on the dendritic morphology. (A)** Model of a reconstructed L5 pyramidal cell. Inhibition was activated with a conductance of *g*_inh_ = 1 nS in the middle of a distal apical dendritic tuft branch and multiple excitatory inputs were distributed uniformly on this branch (enlarged in circle) and activated at 10 Hz. Excitatory synapses near the bifurcation were depressed (cyan circles), whereas more distal synapses were potentiated (red). **(B)** Top: A simplified model emulating the encircled distal branch in **(A)** Excitatory synapses (*E*) were evenly distributed in this modeled branch and inhibitory input (*I*) was placed at either *X* = 0 (orange), *X* = 0.07 (green), or *X* = 0.14 (purple). Bottom: [Ca^2+^]_i_ versus electrical distance from the bifurcation point for the case without inhibition (black) and with inhibition at the three locations depicted at the top. The thresholds for potentiation (*θ*_p_) and depression (*θ*_d_) are marked by horizontal lines. **(C)** Weights for the excitatory synapses as a function of the electrical distance of the synapse from the bifurcation point for the three inhibitory loci [colors correspond to locations depicted in **(B)**]. Location of inhibition determines the transition point between synapses undergoing LTD and LTP. **(D)** The main apical trunk was endowed with an inhibitory synapse at *X* = 0.5 (*I*) and uniformly distributed excitatory synapses (active at 10 Hz). Excitatory synapses at the center of the branch were depressed (cyan region) whereas synapses at both ends were protected (black region). **(E)** Top: A simplified cylindrical model emulating the dendritic branch in **(D)** Bottom: [Ca^2+^]_i_ versus electrical distance in the simplified model. **(F)** Synaptic weights versus electrical distance for the case depicted in **(E)**.

**Figure 4:**
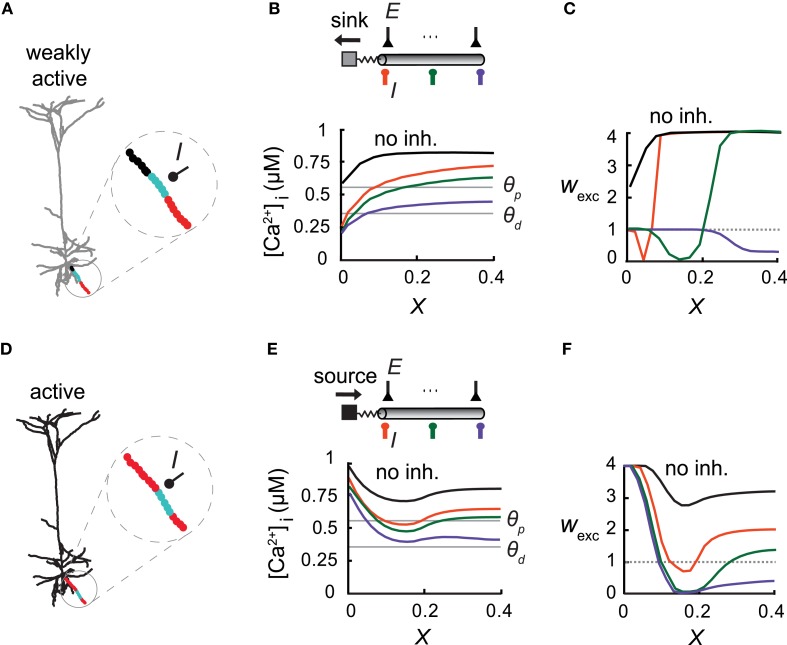
**Overall activity level in the modeled cell modifies the inhibitory effect on excitatory plasticity. (A)** Model of a L5 Pyramidal cell with low synaptic activity throughout the entire cell (2366 uniformly distributed excitatory synapses, each activated at 1 Hz). A basal branch consisting of an inhibitory synapse with a conductance of *g*_inh_ = 1 nS at *X* = 0.5 (*I*) and with multiple excitatory inputs (each activated at 10 Hz). The considerable electrical length of this branch and the significant load at the proximal end allowed for the simultaneous occurrence of all three plasticity states (protected—black, depressed—cyan, and potentiated—red) throughout the branch. **(B)** Top: A simplified model emulating the dendritic branch in **(A)**. Inhibitory input (*I*) was activated at *X* = 0 (orange), *X* = 0.07 (green), or *X* = 0.14 (purple). Bottom: [Ca^2+^]_i_ versus electrical distance from the bifurcation point without inhibition (black) and with inhibition at three different locations (colors corresponding to top figure). Thresholds for potentiation (*θ*_p_) and depression (*θ*_d_) are marked by horizontal lines. **(C)** Weights of the excitatory synapses as a function of distance from the bifurcation point for three loci of the inhibitory synapses [colors as in **(B)**]. **(D)** Same L5 pyramidal cell model as in **(A)** but now the excitatory synapses were simulated at a high rate (10 Hz). Synapses near the soma (*X*=0) switched from a protected state to LTP [red, compare to **(A)**]. **(E,F)** As in **(B,C)**. Synaptic weight distribution is determined not only by the location of inhibition, but by the level of cell activity as well.

**Figure 5:**
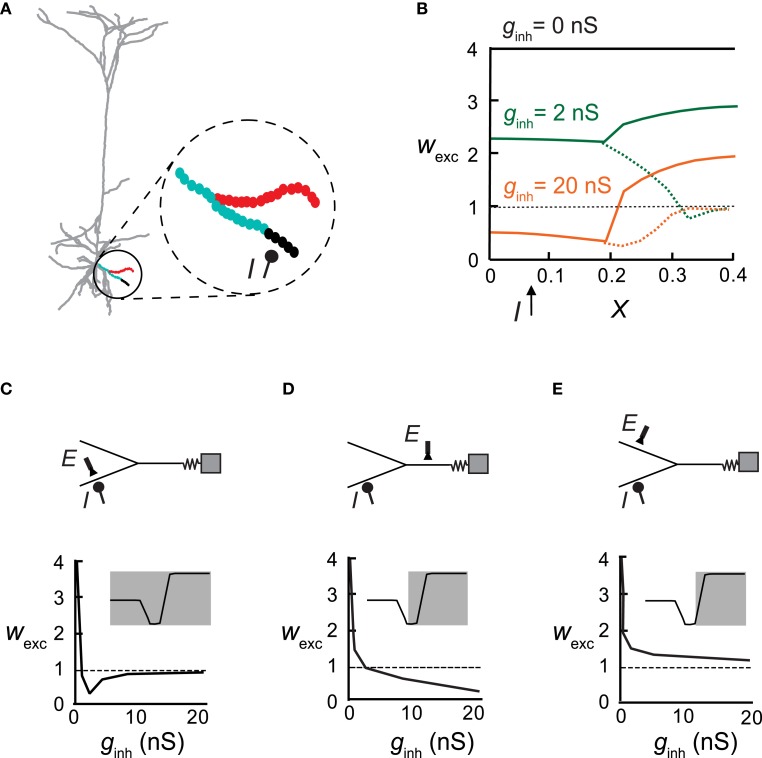
**An inhibitory synapse differentially impacts plasticity in different adjacent branches. (A)** Excitatory synapses (circles in inset) were activated at 10 Hz on a distal basal bifurcating branch (parent and two daughter branches) in the pyramidal cell model. Inhibition (*I*) on one of the daughter branches (at *X* = 0.08 with *g*_inh_ = 5 nS) generated a local protected region (black) whereas LTD ensued at the more proximal region and at the father branch (cyan). Synapses on the sister branch were strengthened (LTP, red). **(B)** Synaptic weights in all three branches versus electrical distance of the synapse from the soma in the absence of inhibition (black), with weak inhibition (*g*_inh_ = 2 nS; green) or with stronger inhibition (*g*_inh_ = 20 nS; orange). Dashed lines represent the inhibited daughter branch. **(C–E)** Top: A reduced model for the distal Y structure in **(A)** with the location of inhibition (*I*) and an exemplar excitatory synapse (*E*). Bottom: The weight of the exemplar excitatory synapse (denoted above) as a function of increasing inhibitory conductance. Insets show the plasticity states that were expressed in the corresponding cases.

A cylindrical cable representing a single dendritic branch was coupled to an isopotential compartment representing the load of the rest of the dendritic tree (Figures 1–5). The soma's diameter, *d*, was 5 μm and its length, *l*, was 5 μm, whereas at the dendrite *d* = 2 μm, and the length, *L*, was 2λ. The load of the soma on the dendritic branch was emulated by setting the dendritic-to-somatic conductance ratio, *ρ*, defined as *R*_in_[soma]/*R*_in_[dendrite], to *ρ* = 0.5. In Figures 2 and 3, the dendritic branch contained 21 excitatory synapses, each with 1 nS AMPA-like and 2 nS NMDA-like kinetics, all activated simultaneously at 10 Hz. In addition, we activated a cluster of steady state inhibitory contacts (with a reversal potential that was equal to the resting potential) with *g*_inh_ = 1 nS. This cluster contained either 5 contacts (Figures 1 and 2B), 10 contacts (Figure 2C), or 15 inhibitory contacts (Figure 2D).

**Figure 6:**
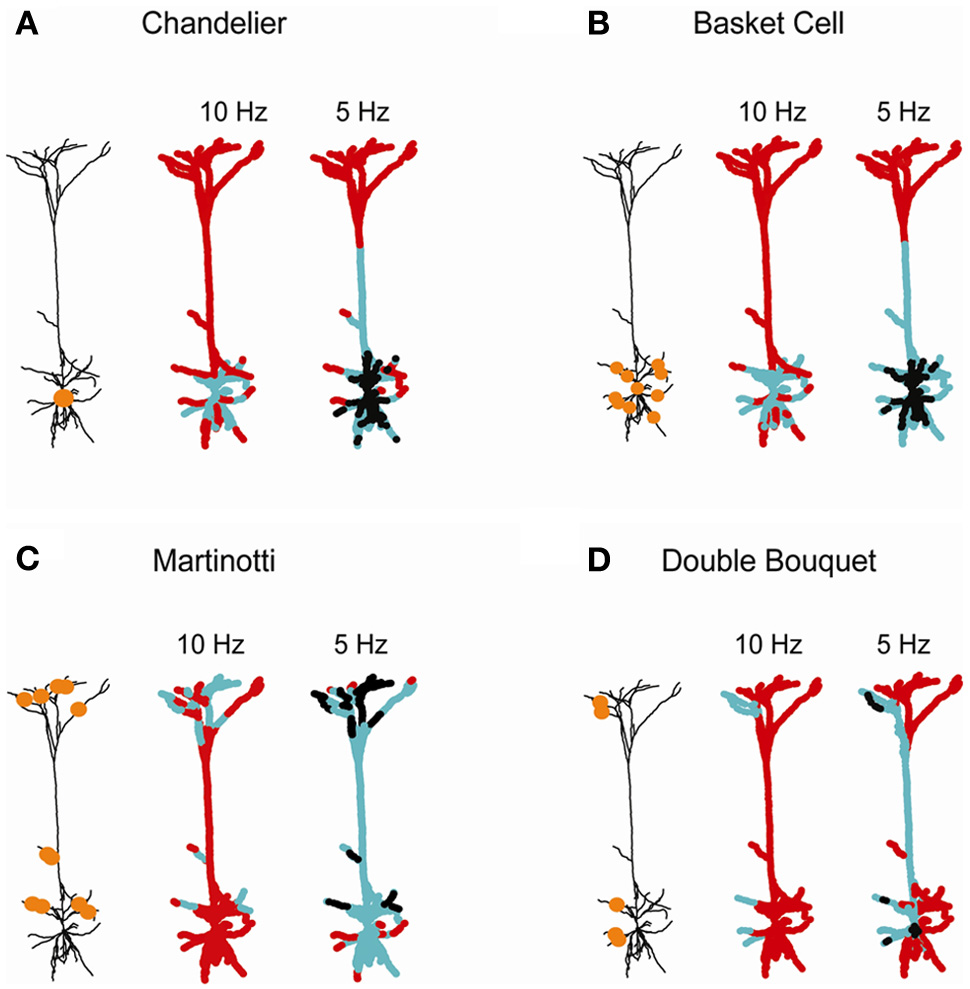
**Domain-specific dendritic inhibition shapes regional excitatory plasticity.** Four inhibitory subclasses impinging on the postsynaptic layer 5 cortical pyramidal cell are modeled: **(A)** Chandelier; **(B)** basket; **(C)** Martinotti; and **(D)** double bouquet. In each case the schemes on the left depict the locations of the inhibitory synapses (orange circles). The conductance of the Chandelier inhibition in **(A)** was *g*_inh_ = 25 nS. In **(B–D)** each inhibitory contact exerted *g*_inh_ = 1 nS [with 25 contacts in **(B,C)** and 10 contacts in **(D)**]. Two thousand three hundred and sixty-six excitatory synapses were uniformly distributed over the modeled tree and randomly activated either at 7 Hz (middle) or at 10 Hz (right). The strength of the excitatory synapses after 60 s of simulation is color-coded, superimposed on the modeled L5 dendrite. Black—protected region (*w*_exc_ = 1), cyan—LTD (*w*_exc_ < 1), and red—LTP (*w*_exc_ > 1).

In each of the three models depicted in Figures 3–5, the diameter and length of the cylindrical cable and *ρ* values matched those of the corresponding detailed model. Electrical length (*X* =L/λ) and *ρ* values were: *X* = 0.14, *ρ* = 0.01 (Figure 3A); *X* = 0.3, *ρ*_1_ = 0.27, *ρ*_2_ = 0.44 (Figure 3B); *X* = 0.4, *ρ* = 0.017 (Figure 4); *X* = 0.4 (of which the child branch *X* was 0.26), *ρ* at parent branch = 0.02 (Figure 5). When the dendritic tree served as a current source for the modeled dendritic branch (Figure 4B), 50 non-plastic excitatory synapses were activated at the somatic compartment at a random rate with a mean of 10 Hz. In Figure 6 synapses were activated randomly at either 7 or 10 Hz.

#### Y-shaped neuron model

This model emulates a distal basal dendritic branch, coupled to two child branches (Figure 5), with diameters of *d*_p_ = 2 μm for the parent branch and *d*_c_ = 1.26 μm for both daughter branches. These diameters were set in accordance with the *d*^3/2^ rule (*d*^3/2^_p_ = d^3/2^_c1_ + d^3/2^_c2_; Rall, 1959). For the parent branch, *L* = 0.1 (in units of λ) and the load of the rest of the tree “hanging” on this branch was emulated by setting *ρ* to 0.02 [as measured in the reconstructed cell: *ρ* = *R*_in_(rest of the cell)/*R*_in_(branch)]. For the daughter branches *L* = 0.25 and *ρ* = 0.01. Each branch contained 11 excitatory synapses, each with 1 nS AMPA-like and 2 nS NMDA-like conductances, activated at an input rate of 10 Hz. A cluster of steady state inhibitory inputs was placed on one of the daughter branches at *X* = 0.08 with *g*_inh_ ranging between 0 and 20 nS and a reversal potential that equaled the resting potential.

#### Reconstructed cell model

A reconstructed L5 pyramidal cell model was used in this work (Figures 3–6), kindly provided by A. Schaefer. Passive properties were as in Schaefer et al. (2003). Maximal compartment length was *l* ≤ 8 μm. Excitatory synapses were uniformly distributed per unit area over the cell surface (Liu, 2004). The synaptic conductance change of a single excitatory input was 1 nS for the AMPA conductance and 2 nS for the NMDA conductance, except when otherwise stated; the input rate was 10 Hz, except for Figure 4A where 1 Hz was used. The number of excitatory synapses differed for each figure in accordance with the different membrane area in each simulated branch (a fixed density of one synapse per 10 μm^2^ was used). We activated 11 excitatory synapses in Figure 3A, 535 excitatory synapses in Figure 3B, and 2366 synapses throughout the entire cell in Figures 4 and 6. Inhibition was modeled as a steady state GABAergic synapse with a reversal potential that is equal to the resting potential. Each inhibitory synapse had a conductance of *g*_inh_ = 1 nS. In Figures 6A–C we placed 25 inhibitory synapses; 10 GABAergic synapses were used in Figure 6D.

### Excitatory synapses and synaptic plasticity

Modeled excitatory synapses had a combination of AMPAR- and NMDAR-dependent conductances, as in Larkum et al. (2009). We modeled the kinetics of the NMDA current by

(1) $$g_{\text{NMDA}}=g_{\text{max}}\left(e^{-t/\tau_{1}}-e^{-t/\tau_{2}}\right)/\left(1+0.25e^{-0.08V}\right)\text{​},$$

where *g*_NMDA_ is the NMDA conductance and *g*_max_ is the peak synaptic conductance, τ_1_ = 90 ms and τ_2_ = 5 ms. The NMDA channels were assumed to be the sole source of Ca^2+^ current, as in Shouval et al. (2002).

AMPA current was modeled with an instantaneous rise time and an exponential decay time constant of 2 ms.

Synaptic plasticity followed a [Ca^2+^]_i_-based learning rule, adapted from Shouval et al. (2002):

(2) $${\dot{w}}_{\text{j}}=\eta ({\left[{\text{Ca}}^{\text{2+}}\right]}_{\text{i}})\;(\Omega ({\left[{\text{Ca}}^{\text{2+}}\right]}_{\text{i}})-w_{\text{j}}),$$

where *w*_j_ represents the strength of synapse *j*, the calcium level is denoted by [Ca^2+^]_i_ and *η* is the learning rate. Synaptic weights were updated at each time step during the simulations. The change in the AMPA and NMDA conductance values was proportional to the change in *w*_j_. According to the learning function, Ω, synaptic plasticity depends on the level of [Ca^2+^]_i_ relative to two thresholds: the threshold for depression (LTD), *θ*_d_, and the threshold for potentiation (LTP), *θ*_p_. When the calcium level is below *θ*_d_, no synaptic modification occurs; if *θ*_d_ < [Ca]_j_ < *θ*_p_, the strength of the excitatory synapse, *w*_j_, is reduced; and for [Ca]_j_ > *θ*_p_, the synaptic strength is increased (Figure 1A). The learning rate, *η*, is assumed to depend on [Ca^2+^]_i_, increasing monotonically with calcium levels (Figure 1B). The Ω function used throughout this paper was as in Shouval et al. (2002):

(3) $$\Omega =0.25+\text{sig}({\left[{\text{Ca}}^{\text{2+}}\right]}_{\text{i}}-\alpha_{2},\;\beta_{2})-\text{0}.\text{25sig}({\left[{\text{Ca}}^{\text{2+}}\right]}_{\text{i}}-\alpha_{1},\;\beta_{1}),$$

where

(4) sig(x, β) = exp(βx)/(1+exp(βx)),

with *α*_1_ = 0.35, *α*_2_ = 0.55 and *β*_1_ = 80, *β*_2_ = 80.

The calcium-dependent learning rate, *η*, was inversely related to the learning time constant, *η* = 1/τ. The functional form of τ was as in Shouval et al. (2002):

(5) $$\tau \;=\;P_{1}/(P_{2}+{\left[{\text{Ca}}^{\text{2+}}\right]}_{\text{i}}P_{3})+P_{4},$$

with *P*_1_ = 0.1 s, *P*_2_ = P_1_/10^−4^, *P*_3_ = 3, and *P*_4_ = 1 s.

In most of the simulations, excitatory synapses received synchronous inputs, except in Figure 6, where input activation times were drawn from a random Poisson process with a mean rate of 7 or 10 Hz.

### Calcium dynamics

Calcium dynamics included a calcium pump:

(6) $$I_{\text{CaP}}={\bar{I}}_{\text{CaP}}\text{/}(\text{1+}(K_{\text{MCaP}}\text{/}({\left[{\text{Ca}}^{\text{2+}}\right]}_{\text{i }}-{\left[{\text{Ca}}^{\text{2+}}\right]}_{\text{basal}}))),$$

where *I*_CaP_ is the Ca^2+^ current flow through the pump (in mA/cm^2^), *Ī*_CaP_ is the maximal current flow through the pump, *K*_MCaP_ is a constant (0.05 mM), and [Ca^2+^]_basal_ is the basal level of intracellular calcium (0.25 μM).

Simulation of longitudinal diffusion between adjacent compartments was via a published NEURON mechanism for calcium diffusion (Carnevale and Hines, 2006). Longitudinal diffusion was modeled by:

(7) $$\frac{d{\left[{\text{Ca}}^{2+}\right]}_{\text{i}}}{dt}=2\times f_{\text{Ca }}(I_{\text{CaP}})\text{ / }d_{s}\text{F},$$

where *f*_Ca_ is the constant fraction of free calcium, *d*_*s*_ is the saturation diffusion coefficient of calcium, and F is Faraday's constant.

## Results

### Inhibition shapes the spatial profile of dendritic calcium concentration

We first explored the interaction between inhibition and synaptic plasticity in the ball-and-stick model (Figure 1C; see “Materials and Methods”). Inhibition was activated on the dendritic compartment, and 21 NMDA- and AMPA-receptor-mediated excitatory synapses excitatory synapses received synchronous input at 10 Hz each, and underwent plasticity according to the learning rule (Equations 2–5). In the absence of inhibition (black line in Figure 1D), the calcium concentration along the dendritic cable varied with location, i.e., [Ca^2+^]_i_ gradually decreased toward the somatic compartment, due to the large sink imposed by the soma. These differences in [Ca^2+^]_i_ along the cable were accentuated when inhibitory input was activated, since local inhibition reduced depolarization and thus decreased calcium levels (Figure 1D, orange line). In accordance with calcium levels, synapses close to the soma and in the vicinity of inhibition were depressed. At the locus of inhibition (*X* = 0.6) the weight of the excitatory synapse was *w*_exc_ = 0 after 60 s of simulation (Figure 1E, cyan; colors corresponding to Figure 1C), whereas further away from inhibition (*X* = 1.6), toward the tip of the dendrite, the excitatory synapses were potentiated (Figure 1E, red). Accordingly, [Ca^2+^]_i_ near the location of inhibitory input was decreased to its basal level (Figure 1E, light cyan trace), whereas near the end of the cable, [Ca^2+^]_i_ levels rose significantly (Figure 1E, light red trace).

### Mapping the local synaptic plasticity rule onto the spatial dendritic domain

Synaptic inhibition, as well as dendritic geometry, affects the spatial dendritic profile of [Ca^2+^] which, in turn, determined the distribution of synaptic weights in the modeled dendrite. The [Ca^2+^]_i_ distribution on the dendritic cable depended on the strength of the inhibitory input. Different levels of GABAergic conductance were simulated using different clusters sizes of inhibitory connections (see “Materials and Methods”). Different spatial profiles of [Ca^2+^]_i_ were generated (Figure 2A), resulting in various distributions of synaptic weights, as depicted following 0, 6, 12, or 60 s of simulation (Figures 2B–D). When inhibition was activated at *X* = 0.6 (as in Figure 1), with a relatively small cluster of GABAergic conductances (total *g*_GABA_ = 5 nS, Figure 2B) synapses in the vicinity of inhibition switched from LTP (black line—without inhibition) to LTD (orange lines), whereas synapses located far from inhibition, toward the end of the cable, remained as potentiated as in the absence of inhibition (black line) after 60 s of simulations (continuous orange line). The spatial profile of *w*_exc_ reflected the shape of the Ca^2+^-dependent synaptic learning rule (Ω function in Figure 1A). With 5 nS (Figure 2B), only the two right-hand states of the Ω function—the LTD and the LTP states—were mapped onto the dendritic spatial profile of *w*_exc_ (Figure 2B, inset). With a larger inhibitory conductance change (*g*_GABA_ = 10 nS, Figure 2C), synapses on the left part of the cable did not undergo plasticity and remained at *w*_exc_ = 1 (protected), since in this case the intracellular calcium level in the dendritic region spanning the soma (*X* = 0) and about *X* = 0.3 was too low to induce plasticity (Figure 2A, green line), and the synaptic weights in this region remained unchanged throughout the simulation (Figure 2C). Near the location of the inhibitory input, excitatory synapses were depressed, whereas further away from the location of inhibition, the excitatory synapses were potentiated. In this case, the entire Ω function was mapped onto the dendritic spatial profile of *w*_exc_ (Figure 2C, inset). Finally, with a very high *g*_GABA_ value of 15 nS, most of the excitatory synapses remained protected, while away to the right of the inhibition, excitatory synapses were depressed (Figure 2D). In this case only the two left-most states of the learning rule curve—protected and LTD—were mapped onto the dendritic cable (Figure 2D, inset).

In the above simulations we used the simpler case of synchronous excitatory inputs. However, the results obtained for this case were also reproduced when the excitatory synapses were activated randomly (not shown). Moreover, to test whether the large gradient of calcium concentration along the cable was the result of the steep transition between LTD and LTP (Shouval et al., 2002), we changed Ω to achieve a more gradual transition between the three states (see “Materials and Methods”). The smoother learning function did not alter the qualitative results obtained above (not shown).

### Spatial distribution of excitatory plasticity in reconstructed dendritic trees

The spatial extent and particular profile of synaptic strengths strongly depended on the location of inhibition and on the cable properties of the dendrites. This is demonstrated in a model of a reconstructed L5 pyramidal cell, focusing on three different dendritic branches (Figures 3 and 4). In each case, we replaced the respective dendritic branch with a model composed of a cylinder (with a diameter and passive properties of that branch) and an isopotential compartment representing the measured load (the boundary conditions) imposed on the modeled branch by the rest of the tree (see “Materials and Methods”). Excitatory synapses were evenly distributed over the dendritic branch and inhibitory input with a conductance of *g*_inh_ = 1 nS was activated in various locations on that branch. We activated synaptic input on single branches, while the rest of the cell remained silent, in order to explore the impact of specific morphological and physiological conditions on synaptic plasticity. Although the conditions of such restricted input are usually not observed experimentally, we found them to be important tools for the understanding of the complex interaction between multiple excitatory and inhibitory inputs as depicted in Figure 6 below. When the inhibitory source was placed in the middle of a small distal apical branch, it induced two distinct plasticity states over different parts of this cable (Figure 3A; red—LTP, cyan—LTD), despite its short electrical length (*L* = 0.14λ). Such dissimilarity between the weights of identical adjacent synapses is the result of the large difference between the boundary conditions at the two ends of the branch, i.e., a large sink (small *ρ*, see “Materials and Methods”) at the proximal end (*ρ* = 0.01 in the modeled tree) and a sealed end at the distal portion of the dendritic branch. This asymmetry gives rise to a large [Ca^2+^]_i_ gradient along the branch in the absence of inhibition (Figure 3B, black). Inhibition impinging on this branch shifted the spatial distribution of [Ca^2+^]_i_ in a manner that depended on the strength and exact location of the synaptic inhibition. With inhibition at the proximal end (*X* = 0, Figure 3C, orange), most of the synapses underwent LTP, whereas synapses near the branch point experienced LTD. Inhibition at the center of the branch (*X* = 0.07, green) resulted in approximately half of the branch's synapses being depressed, while the synapses at the distal part were potentiated. With inhibition at the distal end (*X* = 0.14, purple), all of the branch's synapses underwent LTD. Thus, tuning the magnitude and/or the location of GABAergic dendritic synapses could serve as a mechanism for fine tuning the plasticity profile over the dendritic tree. Surprisingly, excitatory plasticity is tuned by local dendritic inhibition in an extremely precise manner, with micrometer resolution.

The degree of inhibitory tuning of the plasticity profile depended on the morphological properties of the dendrite. For example, in the relatively long main apical trunk (Figure 3D), the current sink at both ends (*ρ*_1_ = 0.27 at the distal end and *ρ*_2_ = 0.44 at the proximal end) lowered [Ca^2+^]_i_ near the branch ends relatively to its center (Figure 3E, black). Consequently, without inhibition, synapses near the branch ends were weakened (LTD), whereas synapses in the middle were strengthened (Figure 3F, black line). The addition of inhibition resulted in LTD at the center (Figure 3D, cyan), whereas synapses at both ends became protected (black). Thus, inhibition lowered [Ca^2+^]_i_ over the whole branch (Figure 3E) and switched the plasticity states of all of the excitatory synapses (Figure 3F). Although this branch was longer than the branch depicted at Figure 3A, the specific location of inhibition did not affect the spatial distribution of synaptic weights (orange, green, and purple lines). The similar boundary conditions at both ends governed the location of the transition point between plasticity states, and inhibition could only determine whether one or two plasticity states would emerge in this branch, and which two states were to exist throughout the branch (LTP/LTD or LTD/protected).

We concluded that the structural properties of the branch, predominantly its boundary conditions (taking into account the excitatory activity), set the spatial profile of calcium concentration along the branch, thus defining the range of possible synaptic weight distribution profiles that could be induced by the activation of inhibition.

### Excitatory activity level modifies inhibitory impact on synaptic plasticity

Inhibition was found to be more effective in carving the synaptic weight distribution at a high spatial resolution when the slope of [Ca^2+^]_i_ along the dendrite was steep, as in the case of terminal branches (with a large sink at the proximal end), and this expression of the three plasticity states within a single branch is expected to occur in long branches with a large load on one end. This is the case depicted in the basal terminal dendrite (Figure 4A; black—protected, cyan—LTD, and red—LTP) where *L* = 0.4λ and *ρ* = 0.017. In this case three plasticity states existed simultaneously along the branch. In a simplified model of this branch (Figure 4B), the large dendritic load, emulated by an isopotential compartment with a corresponding *ρ* value, generated a sufficiently large [Ca^2+^]_i_ gradient (Figure 4B) for inhibition to change the location of both transition points between the three plasticity states along this branch (Figure 4C).

We next examined synaptic plasticity on this single basal branch during the activation of excitatory synapses (2321 non-plastic synapses) over the whole modeled neuron. We uniformly distributed 45 excitatory synapses on this branch and activated them simultaneously at 10 Hz. The rate of excitatory synaptic activity over the entire cell was varied. When excitation was activated on the depicted basal branch alone, the dendritic tree served as a current sink at the proximal end of the basal branch (Figures 4A–C). In contrast, strong activity (10 Hz for all synapses) served as a current source for this basal branch, leading to the strengthening of excitatory synapses at the proximal end (Figure 4D, red). In this particular simulation, in order to avoid saturation of LTP due to very high [Ca^2+^]_i_ along the branch we decreased the peak excitatory synaptic conductance on this branch by a factor of 2. We emulated this high activity state in a simplified “ball-and-stick” model by activating 50 excitatory synapses at the isopotential compartment, each at a rate of 10 Hz input (Figures 4E,F). The synaptic conductance, *g*_max_, of the excitatory synapses in the dendritic compartment was decreased by a factor of 2, as in Figure 4D. The voltage source led to an increase in [Ca^2+^]_i_ near the proximal end of this branch (Figure 4E), resulting in the potentiation of the proximal synapses (Figure 4F), rather than them being protected in the low activity case (Figure 4C). More distal synapses experienced LTD under these conditions (Figure 4F). Thus, both the degree of excitatory synaptic activity, and inhibition strength and location, determined the spatial distribution of the synaptic weights. Therefore, various weight distributions become possible under different conditions of excitatory and inhibitory activity.

### Differential effect of local inhibition on neighboring dendritic branches

Next inhibitory input was placed on a single basal dendrite and excitatory synapses were activated on that same branch and on its parent and sister branches (Figure 5A, inset). We found that the “fate” of plasticity of the excitatory synapses depended on their branch location: for *g*_inh_ = 2 nS, the strength of the excitatory synapses remained unchanged in the immediate vicinity and distal end of the inhibited branch (black), whereas more proximally on this branch, LTD was induced (cyan). The synapses on the parent branch were also depressed but excitatory synapses on the sister branch were potentiated (red). Using an equivalent Y-shaped neuron model (insets in Figures 5C,D and see “Materials and Methods”) we activated inhibition on one of the distal locations (*I*). As *g*_inh_ increased from 2 nS (Figure 5B, green) to 20 nS (Figure 5B, orange), a larger area of the inhibited branch was rendered protected (*w*_exc_ = 1), more synapses switched from LTP to LTD in the inhibited branch and its parent branch, and LTP amplitude decreased in the sister branch.

Figures 5C–E plots the weights of excitatory synapses situated at different loci as a function of increasing inhibitory conductance at a constant location. At the locus of inhibition (Figure 5C), the excitatory synapse reached the non-plastic state very rapidly—low *g*_inh_ was sufficient to reduce calcium levels below the LTP threshold. Above *g*_inh_ = 10 nS, the calcium level was too low to induce plasticity. Parent synapses (Figure 5D) did not switch to the non-plastic stage at any value of *g*_inh_, but rather switched from LTP to LTD. Synapses on the sister dendrite (Figure 5E) were always potentiated, independently of the magnitude of the inhibitory input. Insets depict the portion of the Ω function that is implemented in the depicted locations of the excitatory synapses, as a function of *g*_inh_. In the inhibited branch all three states existed, at the parent only two states were possible (LTP/LTD), and at the sister branch only LTP was found.

### Domain-specific dendritic inhibition and micro domains of synaptic plasticity

Individual inhibitory neurons typically innervate pyramidal cells via multiple synaptic contacts; each class of inhibitory neurons targets different subdomain of the post-synaptic dendritic tree (Markram et al., 2004; Gidon and Segev, 2012). We therefore expanded our analysis to explore the implications of such inhibitory connectivity pattern on excitatory plasticity (Figure 6). Four domain-specific patterns of inhibitory innervation were examined: (1) Axo-axonic (Chandelier) inhibition (Somogyi, 1977; Somogyi et al., 1982; DeFelipe et al., 1985); (2) Peri-somatic (basket cell) inhibition forming multiple inhibitory contacts onto the soma and proximal basal and apical dendrites of the target pyramidal cells (Somogyi et al., 1983; DeFelipe et al., 1999); (3) Distal dendritic inhibition, such as formed by Martinotti cells onto the oblique and apical branches (Wang et al., 2004; Silberberg and Markram, 2007); and (4) Sparse inhibitory connections onto different dendritic regions, such as those formed by double bouquet (DB) inhibitory cells (Somogyi and Cowey, 1981; DeFelipe et al., 1989, 1999).

Axo-axonic inhibition, modeled by 25 inhibitory synapse of *g*_GABA_ = 1 nS onto the initial segment of the axon (Chandelier-like inhibition, Figure 6A), modified synaptic weights in the peri-somatic region. LTD was induced around the soma for higher excitatory activity (10 Hz; middle), while lower activity (7 Hz; right) resulted in LTD in the main apical trunk and a large peri-somatic non-plastic area. The neuronal activity level was critical in determining the span of inhibition's effect on plasticity, since it set the sink level for each dendritic branch. High activity served as a voltage source for each branch, resulting in a localized inhibitory effect on synaptic weights and in the absence of protected synapses. In contrast, a low rate of background synaptic activity generated a sink at each bifurcation point, leading to a decrease in [Ca^2+^]_i_ and lowered synaptic weights at a peri-somatic zone. This result is consistent with the findings of Gidon and Segev (2012), namely that given multiple synaptic contacts, inhibition operates globally, spreading centripetally up to hundreds of micrometers from the location of the inhibitory synapses.

For multiple inhibitory contacts onto the soma and basal branches, simulated by 25 inhibitory synapses with *g*_GABA_ = 1 nS (basket cell inhibition, Figure 6B), the range of inhibitory influence and the degree of plasticity modification was very similar to that of the axo-axonic inhibition for both high and low activity levels in the cell. This result is in accordance with previous findings, showing that due to a centripetal effect, peri-somatic dendritic inhibition generates an almost identical profile of shunt level (namely, the shunting effect of inhibition on the dendrites) over the cell as axo-axonic inhibition (Gidon and Segev, 2012).

Dendritic inhibition targeting both apical and basal branches, simulated by 25 synapses of *g*_GABA_ = 1 nS each (Martinotti inhibition, Figure 6C) enabled modulation of plasticity in both basal and apical micro domains (middle). As the background excitatory rate decreased to 7 Hz (right), the centripetal effect enabled inhibition to affect a substantially wider area, inducing a modification of excitatory synaptic weights throughout the apical tree.

Sparse inhibitory connections onto two dendritic domains, as formed by a single DB inhibitory cell, were simulated by 10 apical and basal contacts, each of *g*_GABA_ = 1 nS (Figure 6D). This type of innervation modified plasticity in a highly localized manner restricted to the source branch of inhibition, at both high and low background activity rate. At a lower rate of activity (7 Hz; right), the extent of weight modification widened, yet it remained much more localized than for the other patterns of inhibitory innervations (Figures 6A–C, right). Thus, sparse inhibitory contacts may override the centripetal effect at low rates of excitatory input. We suggest that fine tuning of synaptic plasticity may be achieved by either a high excitatory activity level or by sparse inhibitory innervations over the dendritic tree.

## Discussion

Long term plasticity of excitatory synapses has been shown to depend strongly on intercellular calcium concentration [Ca^2+^]_i_ (Bear et al., 1987; Lisman, 1989; Artola and Singer, 1993; Neveu and Zucker, 1996; Hansel et al., 1997; Yang et al., 1999; Cho et al., 2001; Cormier et al., 2001). Inhibition affects membrane potential, thereby affecting [Ca^2+^]_i_, via voltage-dependent trans-membrane Ca^2+^-currents (as in the case of NMDA-receptors). Consequently, inhibitory synapses in dendrites are likely to play a key role in regulating the induction of synaptic LTP and LTD in excitatory synapses. The interplay between excitatory plasticity and inhibitory activity in dendrites has never been systematically examined; this was the aim of the present computational study.

Notably, this chain of effects does not imply that the spatial profile of plasticity could be simply deduced from the spatial voltage profile. Indeed, as we have demonstrated in this work, morphological factors, such as branch diameter and boundary conditions, have a marked effect on the spatial profile of [Ca^2+^]_i_, and therefore on plasticity of the respective synapses. Independently of the particular Ca^2+^-dependent plasticity model selected, the effect of the dendritic geometry on [Ca^2+^]_i_ would have a prominent effect on plasticity, and this effect, non-linearly combined with the effect of dendritic cable properties on voltage, was the primary focus of the present study.

We demonstrated that dendritic inhibition could control the weight distribution of excitatory dendritic synapses in a branch-specific manner and at micrometer spatial resolution. This inhibitory control depends on the exact localization and strength of the inhibitory synapses, on the detailed geometry of the dendritic tree and on the level of excitatory activity. We propose that, in addition to its “classical” role in controlling local dendritic excitability (Miles et al., 1996; Gidon and Segev, 2012) and, consequently, the neuron's input-output relationship, dendritic inhibition plays a key role in finely modulating synaptic plasticity in dendrites in a domain-specific manner.

One direct testable prediction of our work, is that the spatial gradient of Ca^2+^ and thus of excitatory synaptic weights is less homogeneous in terminal branches as compared to the more proximal branches. This inhomogeneity is amplified by the activation of synaptic inhibition in terminal branches. By optically measuring the [Ca^2+^]_i_ profile in different dendritic branches, while uncaging local GABA in these branches, the above effect could be substantiated and quantified. It would also be important to demonstrate that local dendritic inhibition could transiently decouple excitatory plasticity from postsynaptic activity. Namely, due to inhibition, the effect of nearby excitatory synapses on the cell's output would be reduced, implying that these synapses are expected to undergo depression (due to a Hebbian mechanism). However, sufficiently strong inhibition would protect these excitatory synapses from undergoing plasticity by suppressing the plasticity inducing signal, namely the [Ca^2+^]_i_. This predicted effect could be measured experimentally in an LTD protocol, combined with GABA uncaging. We expect that synapses in the vicinity of inhibition would not undergo LTD, but rather would retain their original efficacy.

## Branch-specific and domain-specific plasticity control by inhibition

Applying the [Ca^2+^]_i_-dependent plasticity rule for NMDA synapses (Shouval et al., 2002), which was based on the classical Bienenstock-Cooper-Munro (BCM) model (Bienenstock et al., 1982), we found that the plasticity state (LTD, LTP, or protected) and the weight of excitatory synapses may be adjusted by inhibition at a fine spatial resolution. In particular, activation of inhibitory synapses localized at distal dendritic branches typically results in the manifestation of the entire range of plasticity states in these branches, whereby some synapses undergo LTD, others LTP, and the rest remain protected. In other words, strategically placed inhibitory synapses can divide the dendritic tree into a set of separate functional dendritic plastic compartments whereby each branch is subdivided into regions with separate plasticity regimes. Surprisingly, the expression of a wide range of plasticity states usually occurred at the electrically short terminal branches but not in longer and more proximal dendritic branches. In distal branches a steep gradient of the input resistance (*R*_in_) is expected, from very high *R*_in_ at the distal end to a significantly smaller *R*_in_ at the proximal end (Rall and Rinzel, 1973). This results in a steep voltage gradient and thus in a steep [Ca^2+^]_i_ gradient when NMDA-synapses are activated in this branch This [Ca^2+^]_i_ gradient provides the substrate for the fine resolution of plasticity modulation in this branch. Indeed, steep [Ca^2+^]_i_ gradients provides favorable conditions for a localized inhibitory synapse to “expose” the above three plasticity states in a single branch.

Inhibitory neurons contact specific dendritic subdomains of their postsynaptic target cells (Markram et al., 2004). Martinotti and DB neurons contact distal and apical oblique branches of L5 pyramidal cells; basket cells encircle the somatic region and Chandelier cells specifically target the axon (Somogyi, 1977; Somogyi et al., 1982, 1983; DeFelipe et al., 1985). In Figure 6 we modeled the expected impact of a single axon emerging from the above four types of inhibitory neurons. Martinotti and DB cells have a local modulatory effect on synaptic plasticity (Figures 6C,D), whereas a more global effect on excitatory plasticity is expected for basket cell inhibition. When activated, basket inhibition is likely to give rise to LTD in the corresponding peri-somatic excitatory synapses, leaving LTP to distal tuft excitatory synapses (Figure 6B). Interestingly, Chandelier inhibition, which is restricted to the axon's initial segment, has a similar effect to basket cell inhibition on the plasticity of peri-somatic excitatory synapses. The degree of these effects strongly depends on the level of excitatory activity over the postsynaptic dendrite (compare left and right color-coded tree in each frame). In conclusion, different subclasses of inhibitory neurons not only divide their labor in controlling the excitability of (and therefore the output from) particular dendritic subdomains (Gidon and Segev, 2012), but they also share the labor of shaping the plasticity of excitatory synapses in these dendritic subdomains.

## Generality of the results

Several models have been developed with the purpose of creating a unified description of synaptic plasticity under diverse induction protocols. Some models are phenomenological, such as the BCM model (Bienenstock et al., 1982) and the spike-timing-dependent plasticity (STDP) model (Clopath et al., 2010), whereas other models provided an explicit description of synaptic plasticity in terms of biophysical quantities such as CaMKII (Lisman and Zhabotinsky, 2001) or calcium concentration (Shouval et al., 2002; Yeung et al., 2004).

Our results regarding the effects of dendritic inhibition on the plasticity of excitatory dendritic synapses are not confined to the particular Ca^2+^-based learning rule chosen in this work (Shouval et al., 2002); they also hold for other synaptic plasticity models which assume the existence of either a voltage—or [Ca^2+^]_i_—threshold for switching between depression (LTD) and potentiation (LTP). Indeed, the key results obtained in this work were replicated using either intracellular calcium concentration or membrane voltage as the signal for plasticity. Results were also reproduced when we changed the slope of the learning function, making the transition between LTD and LTP less steep (the following parameter values were changed to create the smoother learning rule: *α*_1_ = 0.25, *α*_2_ = 0.85, *β*_1_ = 20, *β*_2_ = 7; results not shown). Furthermore, similar results were obtained when the model included spiking activity at the soma (not shown).

In this study, excitatory synapses were located on dendritic shafts, rather than on dendritic spines (Peters and Kaiserman-Abramof, 1970; Segev and Rall, 1988; Koch and Zador, 1993; Shepherd, 1996; Yuste and Majewska, 2001; Chklovskii et al., 2002). Imaging experiments have shown that spines act as biochemical compartments, restricting increases in Ca^2+^ concentration to individual synapses when the excitatory synapses on the spine are activated (reviewed by Yuste et al., 2000; Grunditz et al., 2008). Inhibitory synapses that innervate such spines (Beaulieu et al., 1992) could thus very specifically control the plasticity of excitatory spine synapses. However, when the inhibitory synapse is activated on the dendritic shaft (as is typically the case)—it effectively shunts the shaft depolarization resulting from the activation of multiple excitatory synapses on this branch. Both the reduces depolarization and the impact of the shaft inhibition spread very effectively into all the dendritic spines covering this branch (Spruston, 2008). Moreover, it has been recently shown by Popovic et al. (2012), using a high-sensitivity *V*m-imaging technique, that there is almost no voltage attenuation from dendritic branches to spine heads. Simultaneous optical measurements of electrical signals from parent dendrites and from different groups of spines with different neck length, enable Popovic and colleagues to demonstrate that the spine neck does not filter membrane potential spreading from the dendrites into the spine heads, even for fast transients. Hence, shaft inhibition would have a marked effect on the plasticity of excitatory synapses located on dendritic spines.

In our study, we modeled synaptic inhibition at steady state. Although the amplitude of a fast transient inhibitory postsynaptic potential (IPSP) attenuates more steeply than that of steady state IPSP, its time integral attenuates very little from the spine base toward the spine head and, in fact, it behaves exactly like a steady state IPSP (Rinzel and Rall, 1974). We also note that cable theory implies that EPSPs attenuate considerably more steeply from the spine head toward the dendritic shaft than do IPSPs in the opposite direction (Segev and Rall, 1988). This implies that shaft IPSPs are expected to strongly affect the membrane voltage (and thus [Ca^2+^]_i_) at the nearby spine heads.

The class-specific strategic placement of inhibition in dendrites parcels the target dendritic tree into a set of compartments that are subject to distinct control of inhibition over synaptic plasticity. Strong activation of inhibitory contacts may “protect” a specific dendritic region from undergoing synaptic plasticity, while these excitatory synapses may continue to impact the cell's output. This situation is unique to the protected state—whereby inhibitory activity keeps [Ca^2+^]_i_ at its basal levels. For other regimes of [Ca^2+^]_i_, inhibition alters both the weight of the excitatory synapse and, therefore, the cell's output.

The neuron's overall activity level is pivotal in determining the range of the inhibitory effect on plasticity. High excitatory dendritic activity renders the inhibitory effect highly restricted to the location of the inhibitory contact. Under this condition, excitatory synapses do not attain the protected state. In contrast, a low background synaptic activity leads to a decrease in [Ca^2+^]_i_ and lowered synaptic weights in a wider area of the dendritic tree. The low [Ca^2+^]_i_ levels enable synapses over a wide dendritic region to be protected from undergoing plasticity. In this condition, the impact of inhibition over excitatory plasticity would operate more globally (Gidon and Segev, 2012).

In this work we focused on studying the basic principles governing the inhibitory effect on the spatial profile of excitatory plasticity in dendrites. We showed, for the first time, how excitatory synaptic plasticity, calcium concentration and synaptic inhibition interact spatially in the dendritic tree. In our work the sole source of Ca^2+^ current was the NMDA channels. It would be challenging to add to this basic analysis more complex cell activity, such as intracellular Ca^2+^ oscillations and local NMDA and Ca^2+^ spikes. Indeed, it was recently shown experimentally that local NMDA spikes in basal dendrites of layer 5 pyramidal neurons are affected by inhibition in a location-specific manner (Jadi et al., 2012). It would be interesting to explore the implications of this effect for plasticity of the corresponding excitatory synapses.

Finally, recent studies have demonstrated activity-dependent structural changes in the innervation pattern of dendritic inhibition (Maffei et al., 2006; Wierenga et al., 2008; Yazaki-Sugiyama et al., 2009; Marik et al., 2010; Keck et al., 2011; Chen et al., 2012; van Versendaal et al., 2012). This mechanism may serve to reshape the spatial landscape of weight distribution of excitatory synapses in dendrites. Indeed, the present work demonstrates that dendritic inhibition is a powerful, location-specific, mechanism for shaping learning, and memory processes in neurons and neuronal networks.

### Conflict of interest statement

The authors declare that the research was conducted in the absence of any commercial or financial relationships that could be construed as a potential conflict of interest.
